# C-Terminal β9-Strand of the Cyclic Nucleotide-Binding Homology Domain Stabilizes Activated States of Kv11.1 Channels

**DOI:** 10.1371/journal.pone.0077032

**Published:** 2013-10-25

**Authors:** Chai Ann Ng, Ying Ke, Matthew D. Perry, Peter S. Tan, Adam P. Hill, Jamie I. Vandenberg

**Affiliations:** 1 Molecular Cardiology and Biophysics Division, Victor Chang Cardiac Research Institute, Darlinghurst, New South Wales, Australia; 2 St Vincent's Clinical School, University of New South Wales, New South Wales, Australia; German Research School for Simulation Science, Germany

## Abstract

Kv11.1 potassium channels are important for regulation of the normal rhythm of the heartbeat. Reduced activity of Kv11.1 channels causes long QT syndrome type 2, a disorder that increases the risk of cardiac arrhythmias and sudden cardiac arrest. Kv11.1 channels are members of the KCNH subfamily of voltage-gated K^+^ channels. However, they also share many similarities with the cyclic nucleotide gated ion channel family, including having a cyclic nucleotide-binding homology (cNBH) domain. Kv11.1 channels, however, are not directly regulated by cyclic nucleotides. Recently, crystal structures of the cNBH domain from mEAG and zELK channels, both members of the KCNH family of voltage-gated potassium channels, revealed that a C-terminal β9-strand in the cNBH domain occupied the putative cyclic nucleotide-binding site thereby precluding binding of cyclic nucleotides. Here we show that mutations to residues in the β9-strand affect the stability of the open state relative to the closed state of Kv11.1 channels. We also show that disrupting the structure of the β9-strand reduces the stability of the inactivated state relative to the open state. Clinical mutations located in this β9-strand result in reduced trafficking efficiency, which suggests that binding of the C-terminal β9-strand to the putative cyclic nucleotide-binding pocket is also important for assembly and trafficking of Kv11.1 channels.

## Introduction

The KCNH gene family encodes three subtypes of voltage-gated K^+^ channels, the *ether-á-go-go* (EAG, Kv10.x), the *ether-á-go-go*-related (ERG, Kv11.x) and the *ether-á-go-go*-like (ELK, Kv12.x) voltage-gated K^+^ channels [1]. These channels contribute to a wide range of physiological processes including neuronal action potential firing [2], phasic contraction in a range of smooth muscles [3], [4], hormone secretion [5]–[7], cell proliferation [8] and cardiac repolarization [9]. Furthermore, dysfunction of Kv11.1 channels is implicated in the heart disease known as long QT syndrome type 2 (LQT2 syndrome), which is associated with an increased risk of cardiac arrhythmias and sudden cardiac arrest [10].

The KCNH subfamily of voltage-gated K^+^ channels share many structural and functional similarities with the KCNA (Shaker, Kv1.x) family of voltage-gated K^+^ channels. They assemble as tetramers, with each subunit containing cytoplasmic N-terminal and C-terminal domains, as well as a transmembrane region containing the voltage sensor domain, composed of four transmembrane helices (S1–S4), and a pore domain, composed of two transmembrane helices (S5–S6) along with an intervening P-loop segment that contains the K^+^ selectivity filter. Yet at a sequence level, even within the pore domain regions, the KCNH family shares more similarity with the cyclic nucleotide gated (cNG) and hyperpolarization-activated cyclic nucleotide-gated (HCN) channel families than they do with the KCNA family of voltage-gated K^+^ channels [1], [11]. In addition, the KCNH family of channels all contain a cyclic nucleotide-binding homology (cNBH) domain in the proximal end of the cytoplasmic C-terminal region, similar to the cyclic nucleotide-binding domain seen in cNG and HCN channels. However, the cNBH domain in the KCNH family lacks the critical arginine residue that binds the phosphate headgroup of cAMP [12] and the β-roll cavity has an overall net negative charge which makes it unfavourable for cAMP binding and thus, in contrast to cNG and HCN channels, the KCNH family of channels do not appear to be directly regulated by binding of cyclic nucleotides [12], [13].

**Figure 1:**
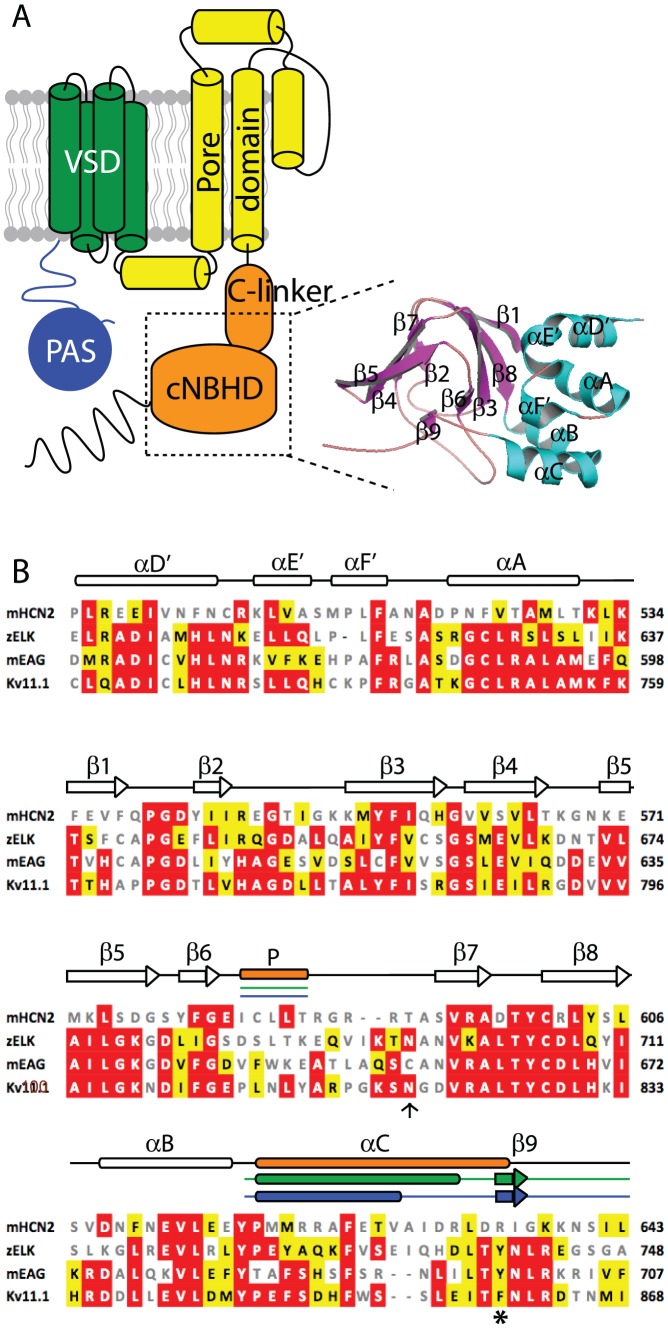
Topology of Kv11.1 channels and sequence analysis of cNBH domains. (A) Topology of Kv11.1 channel showing the intracellular N-terminal PAS domain (blue), transmembrane voltage sensing domain (green), pore domain (yellow) and intracellular C-terminal C-linker and cNBH domains (orange). Inset shows the homology model of the cNBH domain of Kv11.1 generated based on the mEAG crystal structure [13]. (B) Sequence alignment of mHCN2, zELK, mEAG and human Kv11.1 extracted from a Clustalw alignment of the entire family of KCNHx/HCNx/CNGx ion channels. Sequences shown correspond to the dotted box region shown in panel A. Sequence similarity to the Kv11.1 are marked by white text/red box (identical) and black text/yellow box (similar). Non-conserved sequences are in grey. Clear rods and arrows represent the consensus α-helices and β-strands while filled rods and arrows indicate the differences with orange, green and blue representing mHCN2, zELK and mEAG, respectively. The hydrogen bond between asparagine (arrow) and tyrosine (asterisk) in zELK is not observed in the others.

Recently, crystal structures have been determined for the cNBH domain of the zebrafish ELK channel (Kv12.1, [14]), and the murine EAG channel (Kv10.1, [13]). Whilst the structures of these cNBH domains are highly similar to those for the cNBD of murine HCN2 channels, they do show an important but subtle difference. Namely, the most C-terminal α-helix in the HCN2 structure is replaced by an extended conformation with a short β-strand (denoted β9, see Figure 1) composed of three residues that bind to the “cyclic nucleotide-binding pocket”. It has been postulated that this “self-liganded” structure mimics the cAMP bound conformation of the mouse HCN2 cNBD and stabilizes the open state of the mEAG/zELK channels.

In this study we use a combination of experimental and theoretical approaches to investigate the role of the C-terminal β9-strand in the cNBH domain of Kv11.1 channels. Our results show that this β9-strand plays an important role in stabilizing the activated (open and inactivated) conformations of the channel. In addition, we examined the phenotypes of two clinically occurring mutations found in the short β9-strand. Both mutants exhibit impaired trafficking, which suggest that the self-liganded structure is also important for normal assembly and trafficking of Kv11.1 channels.

## Materials and Methods

### Molecular biology

Kv11.1 cDNA (a gift from Dr Gail Robertson, University of Wisconsin) was subcloned into a pBluescript vector containing the 5′ untranslated region (UTR) and 3′ UTR of the *Xenopus* laevis β–globin gene (a gift from Dr Robert Vandenberg, University of Sydney). Mutagenesis of Kv11.1 cDNA was carried out using the Quikchange method (Agilent Technologies, Mulgrave, VIC, Australia) and confirmed by DNA sequencing. WT and mutant channel cDNAs were linearized with BamHI-HF (NEB, Ipswich, MA, USA) and cRNA was transcribed with T7 RNA polymerase using the mMessage mMachine kit (Ambion, Austin, TX, USA).

### Oocyte preparation

Female *Xenopus* laevis frogs were purchased from Nasco (Fort Atkinson, WI, USA). All experiments were approved by the Garvan/St Vincent's Animal Ethics Committee (Approval ID 11/37). Following anaesthetization in 0.17% w/v tricaine, the ovarian lobes were removed through a small abdominal incision. The follicular cell layer was removed by ∼2 hour digestion with 1 mg/ml Collagenase A (Roche, IN, USA) in Ca^2+^–free ND96 solution containing (mM): NaCl 96, KCl 2, MgCl_2_ 1.0 and Hepes 5 (pH adjusted to 7.5 with 5 M NaOH). After rinsing with ND96 (as above, plus 1.8 mM CaCl_2_), stage V and VI oocytes were isolated and stored at 18°C in tissue culture dishes containing ND96 supplemented with 2.5 mM pyruvic acid sodium salt, 0.5 mM theophylline and 50 µg/ml gentamicin. *Xenopus* laevis oocytes were injected with cRNA and incubated at 18°C for 24–48 h prior to electrophysiological recordings.

### Electrical recordings and data analysis

All experiments were performed at room temperature (∼21°C). Two–electrode voltage–clamp (TEVC) experiments were performed using a Geneclamp 500B amplifier (Molecular Devices Corp, Sunnyvale, CA, USA). Glass microelectrodes had tip resistances of 0.3–1.0 MΩ when filled with 3 M KCl. Oocytes were perfused with ND96 solution (see above). A step of +20 mV from the holding potential of −90 mV was applied at the start of each sweep to enable off–line leak–current subtraction and we assumed that the current leakage was linear in the voltage range −160 to +40 mV. Data acquisition and analysis were performed using pCLAMP software (Version 10.2, Molecular Devices, Sunnyvale, CA, USA), Excel software (Microsoft, Seattle, WA, USA) and Prism 6 (GraphPad Software Inc. La Jolla, CA, USA). All parameter values were calculated as mean ± standard error of the mean (SEM) for *n* experiments, where *n* denotes the number of different oocytes studied for each construct.

To measure the voltage dependence of activation, cells were depolarized from a holding potential of −90 mV to voltages in the range −70 to +40 mV for 3 s, before stepping to −70 mV to measure tail current amplitudes [15]. Tail current amplitudes were normalized to the maximum tail current (*I*_max_) and fitted with a Boltzmann expression to derive the V_0.5_ of activation:
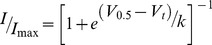
(1)where *I/I_max_* is the relative current, *V*_0.5_ is the half–activation voltage, *V*_t_ is the test potential and *k* is the slope factor. To measure the voltage dependence of deactivation, cells were depolarized to +40 mV for 1 s, to ensure channels were fully activated, then stepped to voltages between 0 and −120 mV for 3 s followed by a step to −70 mV to measure tail currents amplitude [16]. Tail current amplitudes were normalized to the maximum tail current value and fitted with a Boltzmann expression (equation (1) above) to derive the V_0.5_ of deactivation. Rates of deactivation were measured from the hooked tail current traces recorded at voltages in the range −60 to −160 mV, after first depolarizing cells to +40 mV for 1 s. Current traces were fitted with a double exponential component (deactivation only) or a triple exponential (one component for recovery from inactivation and two components for deactivation) [17].

Rates for the recovery from inactivation were obtained from exponential fits to current traces recorded between −20 and −160 mV following a 1 s depolarization to +40 mV. Rates of inactivation were measured using a triple pulse protocol, with cells depolarized to +40 mV for 1 s, repolarized to −90 mV for 10 ms and then depolarized to test potentials in the range +60 and −40 mV. By plotting the rates of recovery and the rates of onset of inactivation, the midpoint of the chevron plot was obtained and the corresponding voltage was taken to represent the midpoint of the voltage dependence of steady-state inactivation [18], [19].

### Trafficking

Constructs for mammalian cell expression have been described previously [20]. Human embryonic kidney cells (HEK293, European Cell Culture Collections) were maintained in Dulbecco modified Eagles medium (DMEM, Invitrogen, NSW, Australia) supplemented with 10% fetal bovine serum (FBS, Sigma, NSW, Australia) in a 37°C incubator with 5% CO_2_. Cells were transfected using the liposome based transfection reagent Lipofectamine 2000 (Invitrogen, as per manufacturer's protocol). Cells, grown in 24-well plates, were harvested 48 hours after transfection. For stable transfection of HEK293 cells, a C-terminal FLAG tagged hERG1a construct in pIRESNeo (Clontech, Clayton, VIC, Australia) was linearized and transfected into low passage number HEK293 cells using Lipofectamine 2000. Stable colonies were selected with 1 mg/mL of Geneticin (Sigma, Castle Hill, NSW, Australia) for 14 days. Expression of hERG1a-FLAG in individual clones was verified by Western blot. Stable clones of hERG1a-FLAG cells were maintained in media containing 0.5 mg/mL of Geneticin (Sigma).

### SDS-PAGE and Western blot analysis

Cell lysates were obtained by first washing the cells 3 times with ice cold Tris buffered saline (TBS, in mM, Tris 50, NaCl 137, pH 7.5), then solubilized in TBS+1% NP40 supplemented with protease inhibitor cocktail (Roche Diagnostics, NSW Australia) and incubated at 4°C for 1 hour on a rotating wheel. Cell lysates were cleared by centrifuging at 16,000 g at 4°C for 30 min, mixed with SDS-PAGE sample buffer, heated to 60°C for 10 min, and resolved by 7.5% SDS-PAGE, before transferring onto nitrocellulose membrane (BioRad, Gladesville, NSW Australia). For quantitative Western blot analysis, the membranes were probed with a mouse monoclonal anti-HA antibody (HA.11, Covance, North Ryde, NSW, Australia) followed by anti-mouse IRDye800 (Li-Cor Biotechnology, Lincoln, NE, USA) and scanned on a Li-Cor Odyssey infrared imaging system. β-actin (Sigma) was used as loading control. The Odyssey application software (version 3) was used to quantify intensities of protein bands.

### Proteinase K digestion assays

HEK293 cells expressing WT or mutant hERG1a constructs were washed with PBS and incubated in proteinase K buffer (in mM, HEPES 10, NaCl 150, CaCl_2_ 2, KCl 10, pH 7.4) with 200 µg/mL proteinase K (Roche, Castle Hill, NSW, Australia) for 45 min at 37°C. Proteinase K was removed by washing cells three times with PBS containing 6 mM phenylmethylsulfonyl fluoride and 25 mM EDTA. Cell lysates were prepared as above for Western blot analysis.

### Coimmunoprecipitation assays

HA tagged hERG1a mutant constructs were transfected into stable hERG1a-FLAG-HEK293 cells. 48 hrs after transfection, cell lysates were prepared as above and 4% of the lysates were kept for analysis of protein expression. The remaining 96% was used for the immunoprecipitation assays. To immunoprecipitate HA tagged proteins, 1.5 µg of HA.11 anti-HA antibody was added to the cleared lysates and incubated for 2 hr at 4°C on a rotating wheel, followed by overnight incubation with 15 µL of protein G Sepharose beads (GE Healthcare, Rydalmere, NSW Australia) to precipitate the immune complex then washed extensively. To elute proteins bound to beads, 20 µl of 2× SDS-PAGE sample buffer was added to the beads followed by heating to 60°C for 15 min. For detecting interactions with hERG1a, the nitrocellulose membrane was probed with HA.11 and a polyclonal anti-FLAG antibody (Covance) followed by anti-mouse IRDye800 and anti-rabbit IRDye680 (Li-Cor Biotechnology). For detecting interactions with hERG1b, horse radish peroxidise conjugated antibodies (anti-HA-HRP and anti-FLAG-HRP, Sigma) were used to probe the membranes.

### Bioinformatics, homology model and MD simulations

The sequence alignment for all 18 members of the human KCNHx/HCNx/CNGx family of ion channels, along with mHCN2, zELK, mEAG were computed using the Clustalw server [21], [22] and the sequence similarities were obtained by taking the sum of identical and similar residues between Kv11.1 and mHCN2, zELK and mEAG. The secondary structure prediction based on the amino acid sequence was performed using the PSIPRED server [23]. The crystal structure of the C-linker cNBH domain of murine EAG channel (PDB ID: 4F8A) was used as the template to generate the homology model of the Kv11.1 cNBH domain using the program Swiss PdbViewer [24] and optimized using SWISS-MODEL Workspace [25], [26]. MD simulations were performed using Amber 12 [27] and the system was set-up as described previously [28]. To generate the AAA mutant, residues Phe860, Asn861 and Leu862 were computationally mutated to alanine. Each system was energy-minimized to remove any clashes and a random seed generator was used to avoid synchronization artifacts [29]–[31] when Langevin dynamics was used during the equilibration and production run. Constraint algorithm SHAKE [32] was used to achieve the 2 fs time-step with Particle mesh Ewald molecular dynamics (PMEMD) to equilibrate the systems first at 310 K followed by 423 K before production run. It has been previously shown that a higher temperature can be used to study the stability of protein by accelerating the unfolding trajectory without affecting the pathway [33]. Using WT as reference, a total of 60 ns of MD trajectories in each case were collected at 10 ps intervals under the constant temperature and volume (NVT) conditions. We did not include the first part of the C-linker (αA′ to αC′) in sequence comparisons, nor in the homology model, as the orientation of this part of the C-linker is significantly different between the X-ray structures from the different channels. Accordingly, a weak restraint was applied to those residues that correspond to the αD′ and to αF′ of the C-linker during the production run, to prevent this part of C-linker from falling apart during the simulations. Analyses of structural (RMSD) and atomic (RMSF) fluctuations were performed using ptraj module for the 60 ns of MD trajectories. Clustering analyses were performed at 10 ns intervals using a radius of 3 Å. The structures with the lowest RMSD to the centroid, at each 10 ns interval, were selected to be the representative structures and visualized in VMD [34]. The Cα contact maps were extracted from the MD trajectories using ptraj module. These maps analyze the contacts gained or lost upon the AAA mutation in the β9-strand. Hydrogen bond analysis was performed using ptraj module with distance and angle cut-off of 3.5 Å and 120°, respectively.

## Results

### Sequence analyses of C-linker and cNBH domain

Functional Kv11.1 channels are tetrametic proteins, with each of the four subunits containing an N-terminal cytoplasmic PAS domain, a transmembrane voltage-sensing domain, a pore domain, and a cytoplasmic C-linker+cNBH domain (Figure 1A). A homologous C-linker+cNBH domain is also present in the cyclic nucleotide regulated HCN and cNG families of voltage gated ion channels. Crystal structures have been solved for the C-linker+cNBH domains from mouse EAG and zebrafish ELK channels [13], [14]. Despite the relatively modest sequence similarity between the C-linker+cNBH domain of HCN2 and mEAG, and zELK structures - spanning from the αD′ of the C-linker domain to the third α-helix of the cNBH domain (denoted αC in Figure 1B) – all three show a remarkable resemblance [13], [14]. There are, however, two deviations between the mHCN2 and mEAG and zELK cNBH domain structures. The first occurs at the P-helix of mHCN2, located between the sixth and seventh β-sheets (β6 and β7 in Figure 1B). This helix is important for the binding of cyclic nucleotides in cNG channels [35]. The second difference can be found at the C-terminus, where the αC of the cNBH domain in mHCN2 is replaced with a shortened α-helix and a short β-strand (β9) in both the zELK and mEAG cNBH domain structures (Figure 1B, green and blue). This β9-strand occupies the pocket into which cAMP is bound in the murine HCN2 structure [13], [14]. Given that the C-linker+cNBH domain of Kv11.1 shows a much greater sequence similarity to zELK (68%) and mEAG (68.5%) than to mHCN2 (43.2%), it is likely that Kv11.1 C-linker+cNBH domain will contain the self-liganded β9-strand structure rather than the extended α-helical conformation [36].

### Functional role of the C-terminal β9-strand in Kv11.1 cNBH domain

**Figure 2:**
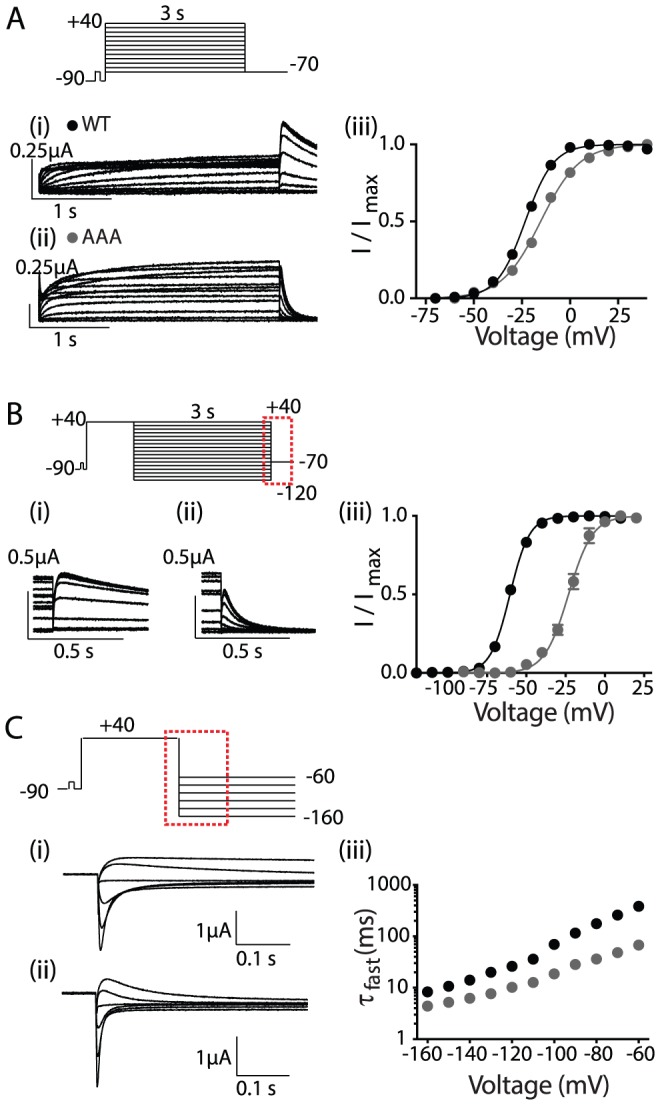
Gating Phenotype of AAA mutant. (A) Family of current traces recorded during a 3 s isochronal activation protocol for (i) WT and (ii) AAA mutant channels. (iii) Isochronal activation curves for WT (filled black circle) and AAA mutant (filled grey circle). The mean V_0.5_ of isochronal activation for the AAA mutant and WT were −15.1±1.0 mV (n = 4) and −23.1±0.4 mV (n = 4), respectively; see Table S1 in File S1. (B) Families of −70 mV tail current traces recorded during a 3 s isochronal deactivation protocol for (i) WT and (ii) AAA mutant. The dotted box in the voltage protocol indicates the portion of the traces shown in the current recordings. (iii) Isochronal deactivation curves for WT (filled black circle) and AAA mutant (filled grey circle). Data are presented as mean ± SEM for n = 4 experiments. The mean V_0.5_ of isochronal deactivation for the AAA mutant and WT were −23.4±1.1 mV (n = 4) and −61.3±0.8 mV (n = 4), respectively. (C) Typical family of current traces recorded between −60 to −160 mV at 20 mV intervals, corresponding to the dotted box in the voltage protocol, used to measure rates of deactivation for (i) WT and (ii) AAA mutant. (iii) Summary of the rates of deactivation for AAA mutant (grey) and WT (black). Data shown as mean ± SEM (n = 4), error bars are within the symbols.

Alanine sidechains have a particularly high propensity to form α-helices [37]. We therefore investigated whether replacing the C-terminal β9-strand in Kv11.1, 860-FNL-862, with alanines (AAA) would impact gating. First, we examined the voltage dependence of channel activation (opening) and deactivation (closing) using the voltage protocols shown in Figure 2A and 2B, respectively (also see methods). Typical families of currents recorded during 3 s depolarization steps to voltages in the range of −70 mV to +40 mV, followed by a short step to −70 mV to measure tail current amplitudes, are shown in Figure 2Ai–ii. By normalizing the peak tail current measured at each voltage to the maximum available current, we can determine the relative fraction of open channels during the preceding voltage step (Figure 2Aiii). The resulting isochronal activation curves can then be fitted with a Boltzmann function to derive the voltage at which one half of the total channels are activated (V_0.5_). The AAA mutant showed only a subtle change in the voltage dependence of activation. Since it can take many tens of seconds for Kv11.1 channels to fully activate at potentials less than 0 mV [15], [38], 3 s isochronal activation curves, such as those shown in Figure 2A, may not reveal the full extent of the differences between AAA mutant and WT channels. We therefore investigated whether the AAA mutant affected isochronal deactivation curves, using the protocol shown in Figure 2B [16]. Cells were depolarized to +40 mV for 1 s to ensure full activation of the channels, then stepped to voltages in the range 0 to −120 mV for 3 s to allow channels to return back to the closed state, before a final short pulse to −70 mV to measure tail current amplitudes. Using this protocol, it is clear that the AAA mutant resulted in a large shift in the V_0.5_ of 3 s isochronal deactivation curves (−23.4±1.1 mV, n = 4, versus −61.3±0.8 mV, n = 4, for WT). The values for the midpoint of isochronal activation and deactivation curves for all mutants investigated in this study are summarized in Table S1 in File S1. It is also apparent from the tail currents recorded at −70 mV for both the isochronal activation (Figure 2A) and isochronal deactivation curves (Figure 2B) that the rate of deactivation for the AAA mutant is much faster than for WT channels. Indeed, the rates of deactivation were ∼4 to 5-fold faster for the AAA mutant compared to WT over the voltage range from −60 mV to −100 mV, and ∼2 to 3-fold faster over the voltage range from −110 mV to −160 mV (Figure 2C). The values for the rates of deactivation at voltages in the range −60 mV to −160 mV for all mutants investigated in this study are summarized in Table S2 in File S1.

**Figure 3:**
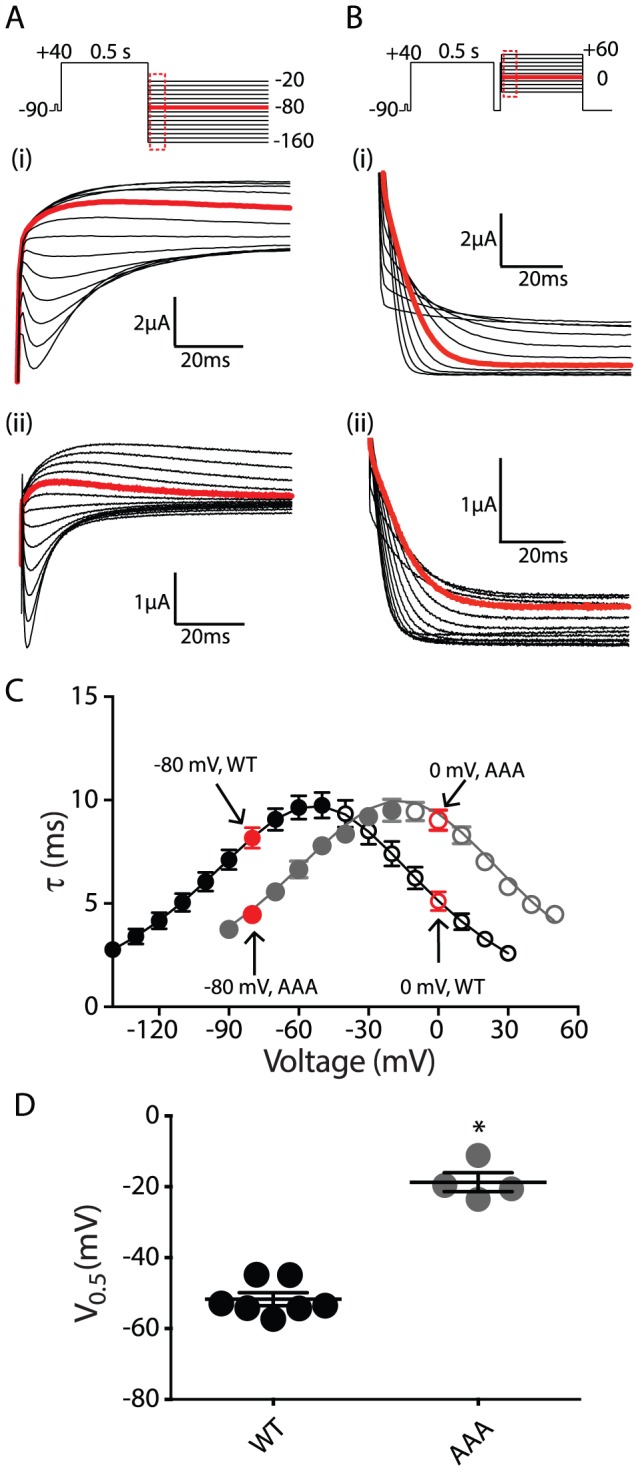
Inactivation phenotype of AAA mutant. (A) Current traces correspond to dotted box in the voltage protocol used to measure the recovery of inactivation for (i) WT and (ii) AAA mutant. Current traces recorded at −80 mV are highlighted to show the faster recovery of inactivation for AAA mutant. (B) Current traces correspond to dotted box in the voltage protocol used to measure the onset of inactivation for (i) WT and (ii) AAA mutant. Current traces recorded at 0 mV are highlighted to show the slower onset of inactivation for the AAA mutant. (C) Summary of rates of recovery and onset of inactivation plotted against voltages between −130 and +50 mV. The data points for −80 and 0 mV are indicated by the arrows. The mid-point of steady-state inactivation for the AAA mutant (grey) is right-shifted by ∼33 mV from WT (black). (D) V_0.5_ of steady-state inactivation for WT (−51.7±1.9 mV, n = 7; filled black circle) and AAA mutant (−18.7±2.7 mV, n = 4; filled gray circle) (*indicates p<0.05 versus WT, ANOVA). Data are presented as mean ± SEM.

The dramatic effect on deactivation gating suggests that the AAA mutant stabilizes the closed state and/or destabilizes the open state. However, at depolarized potentials, Kv11.1 channels predominantly exist in an inactivated state. The assays in Figure 2 do not allow us to distinguish between stabilization of the open state versus the inactivated state. To examine the effect of the AAA mutant on inactivation gating we used a two pulse voltage protocol to derive rates of recovery from inactivation (Figure 3A) and a three pulse protocol to derive rates for the onset of inactivation (Figure 3B). Traces recorded at −80 mV are highlighted in panel A (recovery from inactivation) and at 0 mV in panel B (onset of inactivation). From the mean data of the measured rates of inactivation and recovery from inactivation at voltages in the range −130 mV to +50 mV (Figure 3C) it is clear that the midpoint of inactivation for the AAA mutant is depolarized by ∼33 mV (Figure 3D), indicating that the AAA mutant destabilized the inactivated state relative to the open state of Kv11.1 channels. The values for the midpoint of steady-state inactivation curves for all mutants investigated in this study are summarized in Table S1 in File S1.

### Effects of point mutations in the ß9-strand on Kv11.1 gating

**Figure 4:**
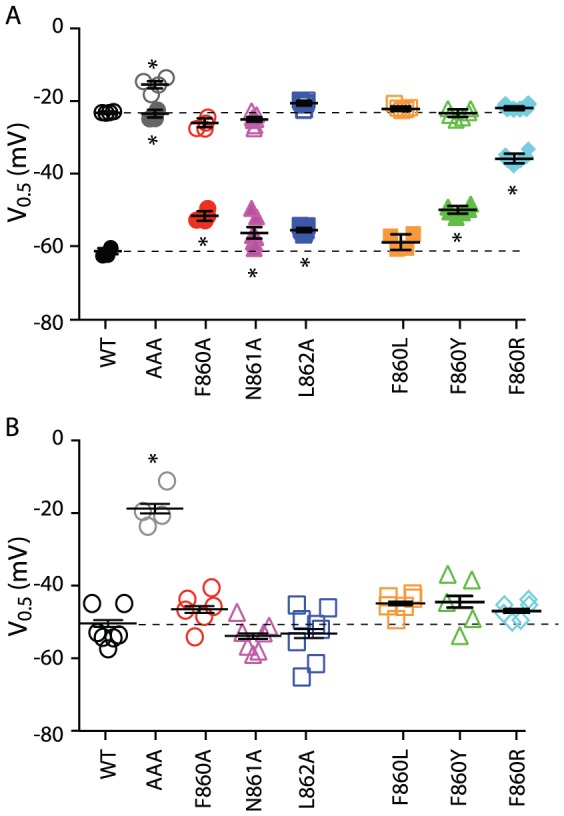
Summary data for individual β9-strand mutants. (A) Scatter plot of the V_0.5_ values for the 3 s isochronal activation (open symbols) and 3 s isochronal deactivation (closed symbols) for WT (black), AAA (grey), F860A (red), N861A (magenta), L862A (blue), F860L (orange), F860Y (green) and F860R (cyan). (B) Scatter plot of the V_0.5_ values for the steady-state inactivation (open symbols) for WT, AAA, F860A, N861A, L862A, F860L, F860Y and F860R (same colour scheme as in panel A). In all panels, the mean and SEM are indicated by horizontal bars and asterisks indicate values that are statistically significantly different to WT (*P*<0.05, ANOVA). The dashed horizontal lines indicate mean values for WT. The values for all mutants are summarized in Table S1 in File S1.

To investigate whether any of the individual residues within the β9-strand played a particularly important role in gating, we individually mutated Phe860, Asn861 and Leu862 to alanine. Data for the voltage dependence of isochronal activation, deactivation and inactivation for all individual mutants are illustrated in Figure 4 and the mean ± SEM data for these measurements are given in Table S1 in File S1. None of the alanine mutants significantly affected isochronal activation but all three produced small but significant shifts in the voltage dependence of deactivation (ANOVA, *P*<0.05) (Figure 4A). The rates of deactivation are also modestly accelerated compared to WT (∼20% in the voltage range −100 to −160 mV, see Table S2 in File S1), which are much less than the ∼4 to 5-fold acceleration seen for the AAA mutant. None of the alanine mutants affected steady-state inactivation (Figure 4B). From these data it is clear that not only do none of the individual alanine mutants have dramatic effects on gating, even the sum of the effect of all three individual mutants is much less than the perturbation caused by the AAA mutant. For example, in the case of the 3 s isochronal deactivation curves, the individual mutants caused a depolarizing shift of +9.7 mV, +5.0 mV and +5.7 mV (a total of +20.4 mV) compared to +37.9 mV for the AAA mutant.

Of the three individual alanine mutants, F860A caused the most significant perturbation to gating. Phe860 is also the least conserved of the three β9-strand residues within the KCNH/HCN/cNG channel families (see Figure 1B) [39]. We therefore examined the effect of mutations to this residue in more detail. We mutated Phe860 to other aromatic side chains, tryptophan and tyrosine, the latter of which is the equivalent residue in mEAG1, hELK1, mELK2 and zELK. We also mutated Phe860 to the bulky hydrophobic residue leucine, and to the charged residue arginine, which is the equivalent residue in mHCN2. Of these mutations, only F860W mutant channels did not express. F860R caused a ∼3-fold acceleration of the rate of deactivation over the voltage range from −60 mV to −100 mV (Table S2 in File S1) and a concomitant ∼25 mV depolarizing shift in the voltage dependence of deactivation (Figure 4A). Much smaller but still significant effects on deactivation were observed for the F860Y mutant, whilst the F860L mutant did not affect the deactivation gating (Figure 4A, Tables S1 & S2 in File S1). Similar to F860A, none of the Phe860 mutants caused significant perturbations to the voltage dependence of inactivation (Figure 4B).

### Do mutations perturb the structure of the β9-strand?

**Figure 5:**
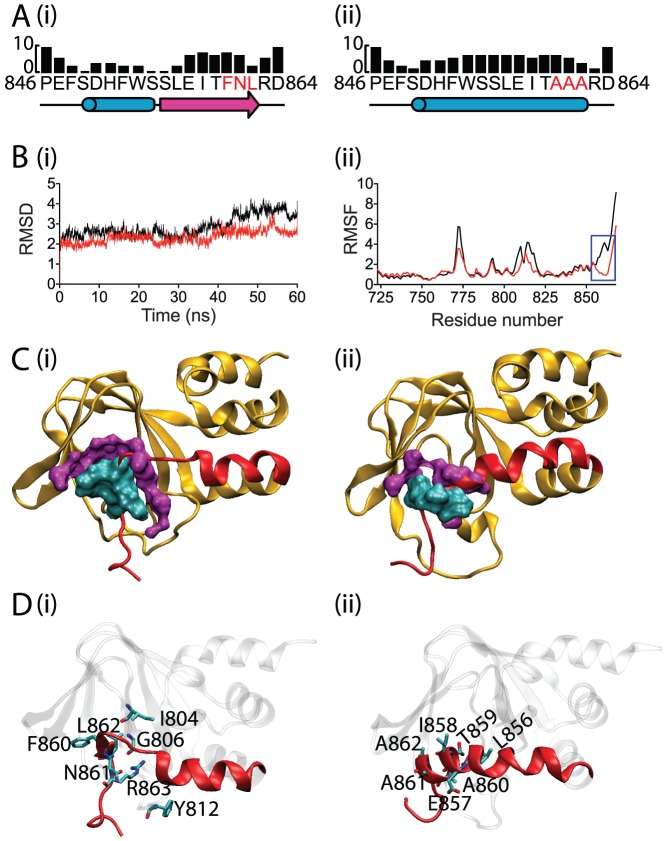
Secondary structure prediction and MD simulations of cNBH domain. Sequence prediction of the cNBH domain around the β9-strand for (A) WT (i) and AAA mutant (ii). (B) RMSD (i) and RMSF (ii) of WT (red) and AAA mutant (black) from the 60 ns of MD simulations. The blue box highlights the most significant difference between WT and AAA mutant in the backbone fluctuation. (C) The structures that have the lowest structural fluctuation to the centroid structure in the most populated cluster from the last 10 ns for WT (i) and AAA mutant (ii). Residues involved in hydrophobic interactions, defined by being within 4 Å of residues 860, 861 and 862 (cyan), are highlighted in magenta for WT (i) and AAA mutant (ii). There are reduced hydrophobic interactions in the AAA mutant. (D) Summary of residues that participate in hydrogen bonds with residues 860, 861 and 862 in WT (i) and the AAA mutant (ii) that are present for more than 5% of the 60 ns of MD simulation (see Table 1 for details).

The electrophysiology data in this study suggest that the β9-strand is required for normal gating of Kv11.1 channels, with the most dramatic affect observed with the AAA mutant. Analysis of the C-terminal end of the Kv11.1 cNBH domain sequence, using the protein structure prediction server (PSIPRED), suggests that the WT protein would form a β-strand (Figure 5Ai). In contrast, the AAA mutant was predicted to form an α-helix (Figure 5Aii). These structural differences could explain the fast deactivation gating observed for the AAA mutant. To investigate whether this prediction was plausible, we used molecular dynamic simulations to study the stability of the β9-strand in the WT cNBH domain, and compared this to the effect of introducing a triplet of alanine residues into the β9-strand of AAA mutant. We used the mEAG C-linker+cNBH domain structure as the template for the Kv11.1 model, as this was the structure with the highest sequence similarity (68.5%) to the Kv11.1 cNBH domain (see Figure 1). Both the WT and the AAA mutant simulations are well equilibrated by 15 ns (Figure 5Bi). The RMSD for AAA mutant is increased slightly after 40 ns due to the formation of stable α-helix at C-terminal. There are some subtle differences in the flexible regions between WT and the AAA mutant (Figure 5Bii) but the most significant difference is in the region around the β9-strand where the AAA mutant shows much higher flexibility than the WT (Figure 5Bii, blue box). By using clustering analysis at 10 ns intervals, we are able to show that the β9-strand remained stable throughout the 60 ns of simulation in the C-linker and cNBH domains of WT channels (Figure S1, left panel). In contrast, the AAA mutant resulted in destabilization of the β9-strand within the first 10 ns and then forms into an α-helix conformation after 40 ns of simulation (Figure S1, right panel), which is consistent with the secondary structure prediction. Further analysis shows the stability of β9-strand conformation (860-FNL-862) observed in WT is mainly maintained by hydrophobic interactions (Figure 5Ci), but also partly by the hydrogen bond between Leu862 and Gly806 that is buried deep in the hydrophobic pocket (Figure 5Di). The hydrogen bond between the backbone amide of Leu862 and the backbone carbonyl of Gly806 was present 93.0% of the time during the 60 ns of MD simulation (Table 1). In contrast, the triple alanine residues (860-AAA-862) in the AAA mutant, which is not able to maintain the β-strand conformation, are not able to form the same interactions with the cNBH domain pocket (Figure 5Cii). Instead, the alanine side chains of the AAA mutant promote the formation of an α-helix conformation (Figure S1 and Table 1). In addition the Cα contacts maps also show gain of contact around the residues 860-AAA-862, which is an indicative of an α-helix formation (Figure S2iii, A). As a consequence there are some loss of direct contacts between residues 860-AAA-862 with the rest of the cNBH domain in AAA mutant, in particular with the residues from 814–823 (loop+β7; Figure S2iii, B) and residues from 775–782 (loop+β3; Figure S2iii, C). Furthermore the interaction between residues 770–776 (β2- β3 loop) and residues 813–822 (loop+β7) is also lost in the AAA mutant (Figure S2iii, D).

**Table 1:** Hydrogen bonds to local (non-bold) and remote residues (bold).

*WT*		*Occupancy*
Phe860-NH	N/A	
Asn861-NH	N/A	
Asn861-HD21	Arg863-O	10.0%
Asn861-HD22	N/A	
Leu862-NH	N/A	

*AAA mutant*		*Occupancy*
Ala860-NH	Leu856-O	18.5%
Ala860-NH	Glu857-O	23.8%
Ala860-NH	Ile858-O	10.6%
Ala861-NH	Glu857-O	18.4%
Ala861-NH	Ile858-O	42.3%
Ala862-NH	Ile858-O	26.0%
Ala862-NH	Thr859-O	43.1%

*WT*		*Occupancy*
**Phe860-NH**	**Ile804-O**	**11.8%**
**Asn861-HD21**	**Tyr812-O(H)**	**7.6%**
**Leu862-NH**	**Gly806-O**	**93.0%**

*AAA mutant*		*Occupancy*
**Ala860-NH**	**N/A**	
**Ala861-NH**	**N/A**	
**Ala862-NH**	**N/A**	

### Phenotype of LQT2 mutants in β9-strand

**Figure 6:**
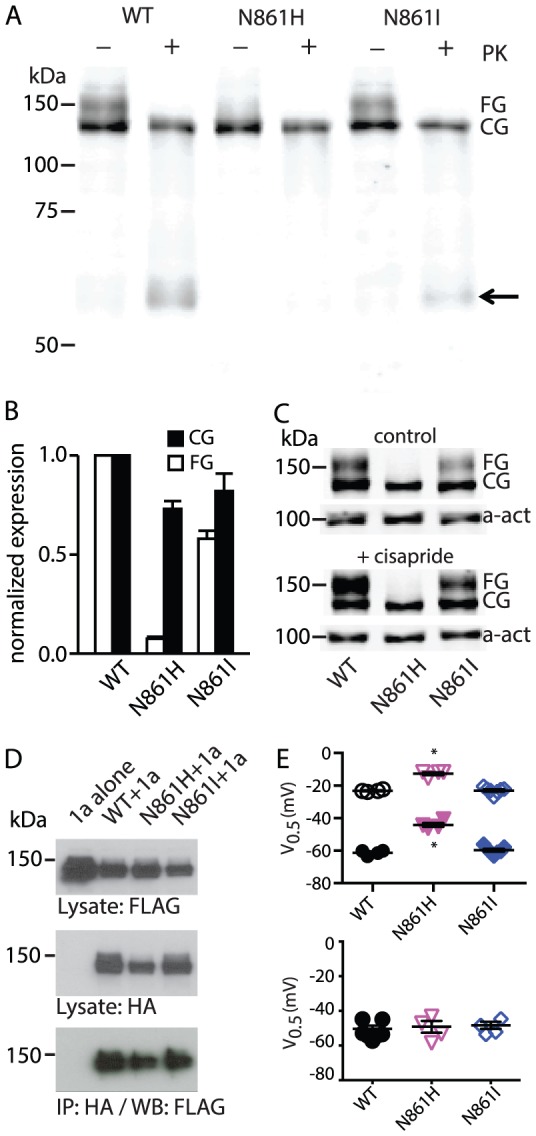
Trafficking assay of LQT2 mutants located within β9-strand. (A) Typical western blot of WT, N861I and N861H mutant channels. WT shows two bands at ∼155 kDa and ∼135 kDa. The ∼155 kDa band disappears following digestion of surface proteins with proteinase K. The N861H mutant shows only a single ∼135 kDa band. N861I contains both ∼155 kDa and ∼135 kDa bands. Arrow indicates degradation band after proteinase K digestion. (B) Normalized expression levels of N861H and N861I relative to WT for the fully glycosylated (∼155 kDa band) and core-glycosylated (∼135 kDa band) proteins. (C) The partially trafficking defective N861I can be rescued by incubation with cisparide whereas N861H was not rescued by cisapride. (D) Co-imunpreciptation of HA-tagged mutant subunits with Flag-tagged WT subunits. (E) Top panel: Summary of 3 s isochronal activation V_0.5_ (open symbols) and 3 s isochronal deactivation V_0.5_ (closed symbols) for WT (black), N861H (magenta) and N861I (blue). Asterisks indicate *P*<0.05 (ANOVA) compared to WT. Bottom panel: Summary of the V_0.5_ of steady-state inactivation for WT, N861H and N861I (same colours as in top panel). Mean data for all mutants are summarized in Table S1 in File S1.

There are two clinical long QT syndrome-causing mutations within the β9-strand that have been reported in the literature; N861I and N861H [40], [41]. As the vast majority of clinical mutants result in trafficking defects rather than gating defects [42], we first investigated whether these mutants affected the trafficking phenotype of Kv11.1 channels. WT Kv11.1 channel protein exhibits two distinct forms, a 135 kDa core glycosylated protein and a 155 kDa fully glycosylated protein, when expressed in HEK293 cells (Figure 6A). Proteinase K digestion of intact cells confirmed that the 155 kDa band corresponds to plasma membrane protein (Figure 6A). In the case of the LQT causing mutations, N861H failed to exit from the ER, exhibiting only the 135 kDa band, while N861I showed some forward trafficking but this was reduced compared to WT (Figure 6B). The trafficking defect in many mutant Kv11.1 proteins can be ameliorated by incubation with cisapride [43], which was the case for N861I but not N861H (Figure 6C). N861H and N861I mutant proteins were able to immunoprecipitate WT Kv11.1 channels (Figure 6D) suggesting that the mutations do not interrupt the formation of tetrameric channels. Expression of N861I mutant channels in *Xenopus* oocytes produced characteristics that were very similar to WT (Figure 6E). Conversely, N861H shifted the isochronal activation and deactivation towards depolarizing potential without affecting steady-state inactivation (Figure 6E).

## Discussion

Recently, crystal structures have been obtained for the cNBH domains of both mEAG and zELK channels [13], [14]. While these structures show a remarkable degree of similarity to the cNBD of HCN channels, their function is clearly very different as they do not bind cyclic nucleotides with high affinity [12] and thus channel gating is not directly regulated by cyclic nucleotides. In this study we show that replacement of the predicted β9-strand in Kv11.1 cNBH domain (860-FNL-862) with alanine residues not only destabilizes the open state relative to the closed state (Figure 2), it also destabilizes the inactivated state relative to the open state (Figure 3). In zELK channels mutations to the β9-strand destabilize the open state [14]. The functional studies in EAG channels are more complex, with different mutants within this motif resulting in either stabilization of the closed state or stabilization of the open state [13]. The simplest explanation of the EAG channel data is that some of the mutants have stabilized the self-liganded structure, whilst others have stabilized the unliganded structure. Overall, our results are consistent with both the ELK and EAG studies, which suggests that in the KCNH family of ion channels the C-terminal β9-strand in the cNBH domain occupies the pocket that otherwise binds cyclic-nucleotides in HCN or cNG channels [44] and that this self-liganded structure stabilizes the open-state conformation of these channels [13], [14]. Our data suggests that the self-liganded state stabilizes the inactivated state over the open state, as well as the open state over the closed state. ELK channels also undergo inactivation [45]–[47]; however, no data have been reported as to whether mutants that disrupt the β9-strand in ELK channels affect inactivation gating in this channel.

Phe860 is the least conserved of the three β9-strand residues. Our more extensive analysis of this residue showed that mutation to arginine, alanine or tyrosine destabilized the open state relative to the closed state, whilst the F860L did not perturb activation/deactivation gating. Based on this data we suggest that the open state is stabilized by the β9-strand interaction with the “cyclic nucleotide-binding pocket” via hydrophobic interactions. It is also worth noting that replacement of Phe860 with the much bulkier tryptophan resulted in channels that did not express, suggesting that the pocket within which the sidechain of Phe860 lies is relatively tight, so that it cannot accommodate the bulkier tryptophan sidechain.

Both protein structure prediction (PSIPRED) and MD simulations suggest that the AAA mutation substantially disrupts the β9-strand, to the extent that it is replaced by an extended α-helix (see Figure 5 and S1), which could explain the dramatic effect seen for the AAA mutant (see Figure 2 and 3). Wray and colleagues had previously shown that mutations of the hydrophobic residues in the β5 segment of the cNBH domain of Kv11.1 channels, to lysine, resulted in a significant acceleration of the rate of deactivation and a depolarizing shift in steady-state inactivation [48], which is very analogous to our results for the AAA mutant. The replacement of buried hydrophobic residues with lysine is of course a fairly dramatic mutation and one cannot exclude the possibility that these mutations resulted in a significant disruption of local folding of the domain. Nevertheless, the data is consistent with the suggestion that the cNBH domain plays an important role in regulating both inactivation and deactivation gating of Kv11.1 channels. This is also reminiscent of recent work from Gustina and Trudeau suggesting that the cytoplasmic N-terminal PAS domain of Kv11.1, a domain well known to be important for deactivation gating [49], also plays an important role during inactivation gating. This does though, raise the possibility that the cNBH domain is just one component of a multi-domain cytoplasmic complex that regulates both deactivation and inactivation gating of Kv11.1 channels. In this context then, our data could be interpreted in one of two ways; either binding of the β9-strand in the pocket of the cNBH domain regulates both deactivation and inactivation gating of Kv11.1 channels, or unbinding of the β9-strand destabilizes the cNBH domain to an extent that it prevents regulation of inactivation and deactivation gating by the putative multi-domain cytoplasmic complex. Given that Phe860 mutants with significantly different physiochemical properties affect deactivation but not inactivation, and MD simulations suggest that the Phe860 mutants do not perturb the structure of the β9-strand, we suggest that the β9-strand plays a direct role in stabilizing the open state relative to the closed state. It is however more difficult to draw more specific conclusions about the role of the β9-strand in stabilizing the inactivated state. Given that the only mutant that significantly affects inactivation, the AAA mutant, also destabilises the β9-strand then it is possible that the β9-strand plays an indirect role in stabilizing the inactivated state by stabilizing the structure of the entire cNBH domain and potentially a multi-domain cytoplasmic assembly. This later explanation would be consistent with the data from Wray and colleagues [48], however clearly it would require more specific investigation if/when structures of the putative cytoplasmic domain assembly become available.

There are two clinical mutants in the β9-strand of Kv11.1 channels that have been reported to be associated with congenital LQT2 syndrome [42], [50]. Like the majority of Kv11.1 missense mutations characterized to date, N861H and N861I resulted in reduced forward trafficking of the full-length channels with 90% and 50% reduction for N861H and N861I, respectively. Trafficking phenotype of N861I in this study is not as severe as reported previously [51]. N861I, however shows normal gating characteristics and so reduced trafficking is still the most likely explanation for the LQT2 phenotype (see Figure 6). The most common mechanism by which mutant proteins are recognized as misfolded and thence tagged for degradation is through exposure of hydrophobic residues, either due to local domain unfolding and/or disruption of domain-domain interfaces [52]. Given that the two neighbouring residues of Asn861 within the β9-strand, Phe860 and Leu862, are both hydrophobic, it is possible that even a subtle mutation at this position could result in local unfolding and exposure of hydrophobic residues. Whilst, our MD simulations do not suggest that N861H/I result in unfolding (data not shown), the role of these mutants in the folding and unfolding of the cNBH domain should be explored further, although this will likely need to await expression and purification of the Kv11.1 cNBH domain.

Despite the profound trafficking defect seen with the N861H mutant in particular, both N861H and N861I were able to co-assemble with WT subunits as shown by co-immunioprecipitation assay (see Figure 6D). This data is consistent with that from Akhaven and colleagues who showed that deletions within the cNBH domain do not disrupt tetramerization [53]. It is also consistent with the crystal structure from the ELK C-linker+cNBH domain showing that the intersubunit interactions occur within the C-linker domain and not the cNBH domain [14].

Very recently, Zagotta and colleagues published the crystal structure of a mosquito ERG cNBH domain that is very similar to that found in mEAG/zELK cNBH domains [54]. The results of their electrophysiology and biochemical studies of Kv11.1 channel are consistent with our findings, which is the β9-strand in the cNBH domain is essential for regulating gating and trafficking of Kv11.1 channels. In addition, our data suggest that the β9-strand plays a direct role in stabilizing the open state and indirectly regulates inactivation gating by stabilizing the structure of the cNBH domain. The importance of the β9-strand for stabilizing the structure of the cNBH domain and hence the quaternary structure of the mature channels is also reflected in the observation that clinically occurring mutants in this β9-strand result in channels that do not traffic and assemble correctly.

## Supporting Information

Figure S1 MD Simulations show destabilization of β9-strand in the AAA mutant.
(EPS)

Figure S2 Cα contact maps showing gain or loss of contacts in the AAA mutant compared to WT.
(EPS)

File S1 Contains: Table S1. Summary of V_0.5_ of 3 s isochronal activation, 3 s isochronal deactivation and steady-state inactivation. Table S2. Summary of the fast component of the rates of deactivation over the voltage ranges of −160 to −60 mV
(DOC)
