# Supplementary material for: C-Terminal β9-Strand of the Cyclic Nucleotide-Binding Homology Domain Stabilizes Activated States of Kv11.1 Channels
Source: PLoS One. 2013 Oct 25;8(10):e77032. doi: 10.1371/journal.pone.0077032 (PMC3808384; doi:10.1371/journal.pone.0077032)
Supplement: File S1 — Contains: Table S1. Summary of V0.5 of 3 s isochronal activation, 3 s isochronal deactivation and steady-state inactivation. Table S2. Summary of the fast component of the rates of deactivation over the voltage ranges of −160 to −60 mV (DOC) [file pone.0077032.s003.doc]

**Table S1: Summary of V_0.5_** of 3 s isochronal activation, 3 s isochronal deactivation and steady-state inactivation.

|  | V_0.5_ 3 s isochronal activation |  |  | V_0.5_ 3 s isochronal deactivation |  |  | V_0.5_ Steady-state inactivation |
| --- | --- | --- | --- | --- | --- | --- | --- |
| WT (4) | −23.2±0.4 mV |  | WT (4) | −61.3±0.8 mV |  | WT (7) | −51.7±1.9 mV |
| AAA (4) | **−15.5±1.0 mV** |  | AAA (4) | **−23.4±1.1 mV** |  | AAA (4) | **−18.7±2.7 mV** |
| F860A (4) | −25.9±1.2 mV |  | F860A (4) | **−51.6±1.4 mV** |  | F860A (6) | −46.5±1.9 mV |
| N861A (7) | −25.3±0.6mV |  | N861A (7) | **−56.3±1.6mV** |  | N861A (7) | −53.8±1.6mV |
| L862A (9) | −20.5±0.7 mV |  | L862A (8) | **−55.6±0.6 mV** |  | L862A (8) | −53.2±2.5 mV |
| F860L (5) | −22.5±0.7 mV |  | F860L (4) | −59.6±2.2 mV |  | F860L (6) | −45.3±1.0 mV |
| F860Y (6) | −23.7±1.1 mV |  | F860Y (6) | **−50.6±1.1 mV** |  | F860Y (5) | −44.8±3.1 mV |
| F860R (6) | −22.3±0.6 mV |  | F860R (6) | **−36.3±1.3 mV** |  | F860R (6) | −47.3±1.0 mV |
| N861H (4) | **−12.7±0.9 mV** |  | N861H (4) | **−44.2±1.2 mV** |  | N861H (4) | −50.5±3.4 mV |
| N861I (7) | −23.0±0.7 mV |  | N861I (7) | −59.8±0.9 mV |  | N861I (4) | −49.7±2.0 mV |

The number of independent experiment is indicated next to the constructs studied. Data is presented as mean ± SEM. *P*<0.05 versus WT using one-way ANOVA Dunnett’s test are highlighted in bold text.

**Table S2: Summary of t**he fast component of the rates of deactivation over the voltage ranges of −160 to −60 mV.

|  | WT (4) | AAA (4) | F860A (4) | N861A (7) | L862A (9) | F860L (5) | F860Y (6) | F860R (7) |
| --- | --- | --- | --- | --- | --- | --- | --- | --- |
| −160mV | 8.3±0.6 | 4.4±0.2 | 7.4±0.4 | 7.0±0.5 | 6.6±0.5 | 9.6±1.2 | 7.6±0.6 | 6.1±0.3 |
| −150mV | 10.7±0.7 | 5.2±0.2 | 9.2±0.5 | 8.9±0.4 | 9.2±0.4 | 11.2±1.2 | 9.4±0.8 | 7.3±0.3 |
| −140mV | 14.1±0.7 | 6.3±0.3 | 11.5±0.6 | 11.5±0.6 | 11.8±0.4 | 15.9±1.9 | 12.1±1.0 | 8.9±0.4 |
| −130mV | 20.0±1.8 | 7.7±0.4 | 14.6±0.7 | 15.6±0.6 | 16.0±0.6 | 20.9±2.1 | 16.0±1.5 | 11.2±0.8 |
| −120mV | 26.2±2.4 | 10.2±0.2 | 19.7±0.7 | 21.4±1.2 | 22.4±1.2 | 28.4±2.7 | 21.2±2.6 | 15.1±0.5 |
| −110mV | 36.2±3.4 | 12.7±0.3 | 25.4±1.5 | 31.9±1.8 | 30.3±1.0 | 39.9±4.9 | 32.9±4.4 | 19.9±0.7 |
| −100mV | 60.1±9.8 | 18.6±1.8 | 39.4±1.2 | 45.5±3.2 | 39.8±2.4 | 89.5±33.3 | 42.6±3.2 | 23.0±4.3 |
| −90mV | 116.2±12.2 | 28.4±2.5 | 81.6±11.5 | 86.9±9.7 | 107.0±3.4 | 125.9±16.5 | 83.9±7.2 | 43.2±5.1 |
| −80mV | 176.6±20.6 | 36.3±0.9 | 121.1±12.6 | 149.5±14.6 | 155.4±4.3 | 188.9±28.3 | 120.8±8.7 | 51.9±6.5 |
| −70mV | 260.0±30.2 | 48.2±3.0 | 191.3±15.0 | 251.4±26.9 | 253.6±10.0 | 268.7±35.8 | 190.2±12.7 | 86.0±4.2 |
| −60mV | ­383.6±42.3 | 68.0±4.1 | 287.1±29.1 | 349.2±27.8 | 390±20.8 | 439.5±69.1 | 295±27.5 | 127.3±9.0 |

The number of experiment is indicated next to the name of the constructs studied. The unit for the rates is msec and data is presented as mean ± SEM.
